# New methodology for specific inhalation challenges with occupational agents

**DOI:** 10.1186/1465-9921-11-72

**Published:** 2010-06-09

**Authors:** Simon Caron, Jean-Christian Boileau, Jean-Luc Malo, Simon Leblond

**Affiliations:** 1 SCL Medtech Inc. Montreal, Canada; 2 Department of Chest Medicine, Sacré-Cœur Hospital, Montreal, Canada

## Abstract

**Background:**

Inhalation challenges are used for diagnosing occupational asthma (OA). The initial methodology consisted of a "realistic" exposure without monitoring nor controlling exposure. Our aim was to design an equipment, called the GenaSIC, that allows the generation of various agents regardless of the formulation and to assess the feasibility of its use in patients investigated for OA.

**Results:**

GenaSIC can generate lactose, flour, malt, isocyanates, formaldehyde and N-butyl acetate with precise and fairly stable concentrations. Using N-butyl-acetate as a control agent and real time measurement, we show that normal breathing has a negligible effect on the concentration. We exposed forty-four different subjects to a control agent and/or to a suspected occupational agent. Nineteen of the subjects were only exposed to N-butyl acetate as a control agent without experiencing any significant irritant effect (no significant changes in spirometry thereafter). Eight subjects who were exposed to both N-butyl acetate and formaldehyde did not show significant reactions. Seven subjects were exposed to dry particles (flour in six instances, malt in the other) and five showed immediate asthmatic reactions which changes in FEV1 from 20% to a maximum of 28%. Finally, ten subjects were exposed to isocyanates, four of whom showed a positive reaction, including one subject with immediate maximum changes in FEV1 of 22%.

**Conclusion:**

GenaSIC offers the possibility of reliable and safe exposures to dry particles, formaldehyde and isocyanates in the investigation of OA.

## Introduction

Occupational asthma (OA) is a type of asthma caused by the workplace. The most common type of OA is the one that occurs after a latency period and that is caused by an acquired sensitization to an agent present in the workplace. Because of the considerable health and socioeconomic consequences [1], it is mandatory to confirm the diagnosis with objective testing. Several means of confirming the diagnosis have been proposed, all of these in a stepwise approach [2,3]. The most precise way of confirming OA is by exposing the worker to the suspected agent and closely monitoring any functional and inflammatory changes. This can be done in the workplace [4] or the exposure can be reproduced in a hospital laboratory in a "realistic way" as originally proposed by Pepys [5].

Because the realistic approach can lead to erratic exposures if the administered concentration or dose of the agent is not carefully controlled [6,7], with the threat of considerable and immediate asthmatic reactions [8], closed-circuit apparatuses have been proposed. This type of exposure in which the concentration of the agent can be continuously monitored and determined during exposure allows to control precisely the dose of inhaled suspected occupational agent. This presents definite safety advantages, limiting the importance of immediate reactions [9]. As in non-specific inhalation challenges, the maximum fall in FEV1 should be limited to 20 to 30% during these tests, a threshold that does not present risk and at which the patient does not experience intolerable symptoms. It is more difficult to control the importance of late reactions [9], but use of this equipment also offers safety advantages over the realistic approach. Moreover, contrary to immediate reactions which are abrupt, late reactions take sufficient time to develop allowing for pharmacological intervention to limit and efficiently treat the asthmatic reaction.

The main limitation of existing apparatuses is that they are incapable of generating all types of agents that can exist as dry particles, wet aerosols and vapors. Therefore, we designed a piece of equipment for specific inhalation challenges with occupational agents that can allow for all types of generation within the same instrument. We also assessed the feasibility of its use by hereby presenting the results of challenges (stability of concentrations, bronchial reactions) undergone by 44 subjects. Since one of the main advantages of the closed-circuit apparatuses is to be able to control with high precision the quantity of inhaled substance, we did several quality tests on the GenaSIC. We specifically studied its stability during the time of a generation and also its reproducibility.

## Material, Subjects and Methods

**Figure 1:**
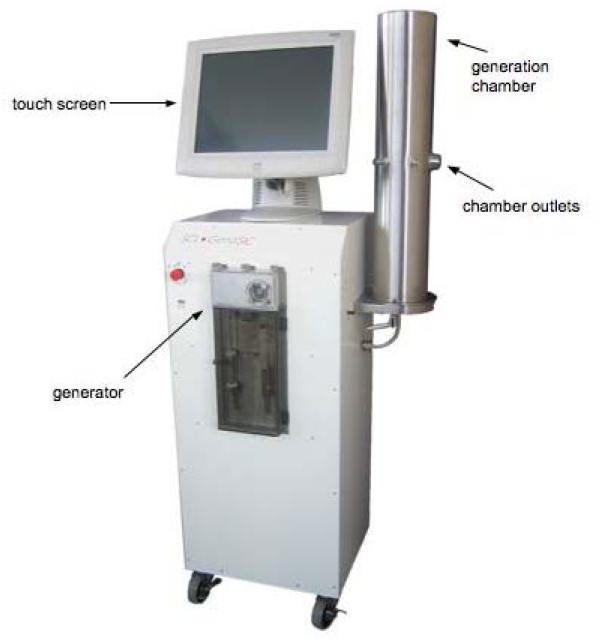
**Key features of the SCL•GenaSIC™**.

The GenaSIC (see Figure 1) is a closed-circuit aerosol generation chamber; i.e., it enables continuous generation of low and stable concentrations of agents, dust or aerosols in an airtight enclosure with controlled atmospheric conditions. This equipment builds upon a first generation of closed-circuit generation systems jointly developed by the Institut de recherche Robert-Sauvé en santé et en sécurité du travail and the Sacré-Coeur Hospital in Montreal in the late 1980s and early 1990s [6,7]. These devices proved to be effective in delivering controlled doses of dusts, vapours or isocyanates. The GenaSIC renders this technology more versatile by merging all three generation capabilities into a single device.

**Figure 2:**
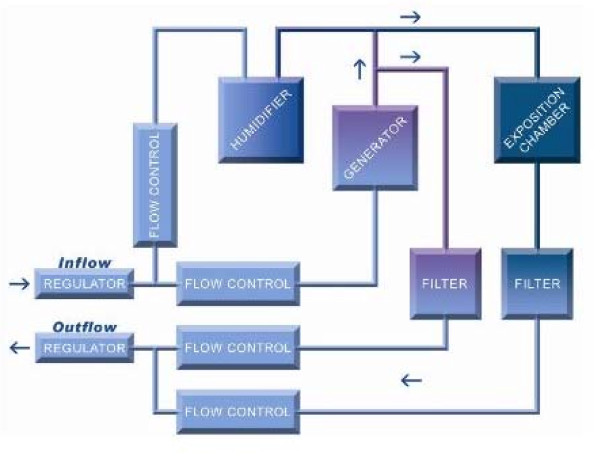
**Generation process of the SCL•GenaSIC™**.

The GenaSIC is capable of aerosolizing dusts, liquids or vapours continuously while maintaining stable concentrations between 0.001 mg/m^3 ^and 1000 mg/m^3 ^depending on the physical attributes of the product. The basic generation process is as follows: the agent being generated is aerosolized by a generator - a syringe-like injector - and brought to the desired concentration by dilution using multiple computer-controlled flow meters. The patient is then exposed at the chamber level where a known and stable concentration circulates at all times. This process is shown in Figure 2.

The GenaSIC's generation capabilities were first validated in a laboratory setting using both sampling and direct measurement instruments. For the formaldehyde and 1.6 HDI, the validation methodology was as follows: for each concentration, direct measurements were taken at fixed intervals (between one second and one minute, depending on instrument used) for the length of the generation period. Simultaneously, five samples were taken over the generation period to ensure an absolute measure of mean concentration. Two different methods were used to reduce potential errors of measurement. For N-butyl acetate, the sampling method was performed and for lactose, we used a direct measurement. To improve our error analysis, supplemental tests were done using real time measurement on some liquid aerosol (N-butyl acetate, formaldehyde, toluene). We could therefore observe any instant variation of concentration and investigate possible sources of errors. Particularly, we have analyzed the effect of breathing on the concentration in the generation chamber. We also have repeated the same set of experiments many times during two weeks to test the reproducibility of the GenaSIC.

Forty-four subjects who underwent specific inhalation challenges between September 2007 and April 2009 were included in the validation process. These subjects were all consecutive patients for whom the cause of OA was one or another of the occupational agents that could be generated using GenaSIC. There was therefore no selection of patients and there was no instance of refusal. All these subjects were investigated for possible OA with standardized procedures that included baseline spirometry [10], responsiveness to methacholine [11] and examination of induced sputum [12]. Specific inhalation challenges were performed as described [13]. Briefly, baseline FEV1 should be ≥ 2 L and variability of FEV1 on the control day should not exceed 10%. In all instances where testing was negative after exposing the subject using the new equipment (no significant changes in spirometry, responsiveness to metacholine and induced sputum cells), the subject was exposed in a "realistic way" [5] for two hours using the same suspected occupational agent. For the realistic exposure to isocyanates, concentrations are monitored and generally do not exceed 20 ppb. In this study, concentrations were not monitored in the case of the realistic exposure to other agents. The control products used were: 1) lactose, if the causal agent was in a dry aerosol; 2) N-butyl acetate, if the causal agent was in an aerosol or vapor form.

The project was accepted by the Ethics Committee of the Sacré-Coeur Hospital and all participants signed an informed consent.

## Results

### Real Time Measurement

**Figure 3:**
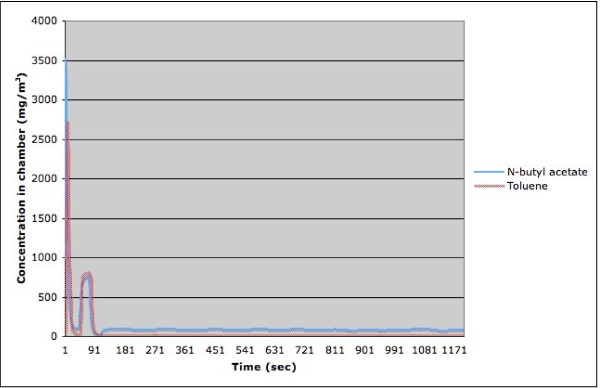
**Chamber concentration variation during typical runs of N-butyl acetate and toluene**.

Although the main indicator of exposition is given by the mean concentration, it is important to ensure that the patient is never subjected to extremely high transient concentration of the suspected occupational agent. For that purpose, we performed a real time measurement of the concentration in the chamber using a ppbRAE 3000, a device based on a photoionization detector, which can produce a new reading every second. We measured N-butyl acetate and toluene and formaldehyde. From the results obtained with these substances, we could calculate the time required to reach a relatively stable concentration and observe the effect of a patient's breathing on the stability of the chamber concentration. At the start of a generation, an extra amount of substance is injected into the chamber when the syringe-like injector is moved into position, creating a very high concentration burst. This is illustrated in Figure 3, which represents the chamber concentration variation during typical runs of N-butyl acetate or toluene. Before letting the patient breath the air in the chamber, we ensured that the initial concentration burst was over. For the liquid aerosols that we tested, the initial concentration burst was always less then 3 minutes. However, in some tests, it has taken slightly longer for the flow in the chamber to stabilize. Table 1 gives the measured time required for the concentration to stabilize in the chamber for five different sets of experiments: three with N-butyl acetate and two with toluene. This suggests that a waiting time (burn-in period) of five minutes is enough to reach the concentration equilibrium in the chamber for the generation of aerosol from liquid that vaporize well, except for very low concentrations, were a waiting time of 10 minutes is more appropriate.

**Table 1:** Time required to reach a steady-state concentration in the chamber

Substance	Targeted concentration (mg/m^3^)	Number of runs	Required burn-in time (sec)			
			Mean	SD	Max	Min
n-butyl acetate	8.5	18	218	104	477	101
	65	20	129	9	150	111
	370	17	120	6	132	108
						
toluene	10	6	153	74	300	111
	150	6	126	12	150	118

**Figure 4:**
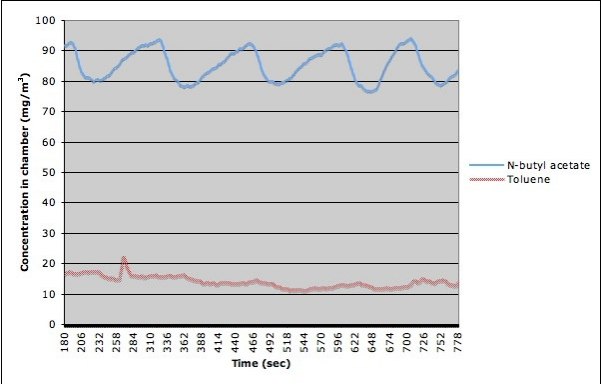
**Concentration of N-butyl acetate and toluene after stabilization of the chamber**.

The initial concentration burst is followed by a stabilization of the chamber concentration. Figure 4 presents the data of the same two experiments as the ones depicted in Figure 3, except that the initial concentration burst was excluded. We can see that both the concentration of the toluene and the one for N-butyl acetate oscillate at a frequency close to 0.008 Hz (close to one oscillation every two minutes). Multiple experiences showed that the frequency of the oscillation did not depend on the concentration of the substance, but rather on the rate of the flow of air through the chamber. We also performed one run with formaldehyde. In this case, the burn-in period is approximately 100 seconds.

### Mean concentrations

The GenaSIC's generation capabilities were validated in a laboratory setting by sampling methods and/or direct measurement instruments. Table 2 presents detailed results of measurements taken during validation runs for N-butyl acetate, formaldehyde, 1.6 HDI and lactose. The main objective was to see if the concentration was similar at the start (after the burn-in period), in the middle and at the end of the generation.

**Table 2:** Targeted and achieved concentrations of generated products during run-in testing

Run-in testing							
Product	Targeted concentration		Obtained concentration				
			Mean	Median	SD	Max	Min
Concentration validated by sampling							
N-butyl acetate	150 mg/m^3^		148	150	5	150	140
N-butyl acetate	250 mg/m^3^		242	240	4	250	240
N-butyl acetate	320 mg/m^3^		322	320	8	330	310
							
Formaldehyde	0.65 mg/m^3^		0.65	0.66	0.05	0.7	0.56
Formaldehyde	1.0 mg/m^3^		1.07	1.07	0.08	1.2	0.98
Formaldehyde	1.7 mg/m^3^		1.69	1.65	0.08	1.82	1.64
							
Isocyanates (1.6-HDI)	0.05 mg/m^3^		0.05	0.06	0.01	0.07	0.03
Isocyanates (1.6-HDI)	0.10 mg/m^3^		0.09	0.09	0.01	0.11	0.08
Concentration validated by direct measurement instrument							
Formaldehyde	0.65 mg/m^3^		0.63	0.63	0.08	0.87	0.36
Formaldehyde	1.0 mg/m^3^		1.07	1.06	0.1	1.94	0.74
Formaldehyde	1.7 mg/m^3^		1.43	1.43	0.04	1.58	1.31
							
Isocyanates (1.6-HDI)	7.2 ppb		6.4	7.0	0.1	7.0	5.0
Isocyanates (1.6-HDI)	14.6 ppb		12.6	13.0	0.5	13.0	12.0
							
Lactose	<10 mg/m^3^		7.9	7.9	0.9	11.50	5.20
							
**Challenges**							
Lactose	<5 mg/m^3^	18	3.25	3.05	0.87	5.45	1.75
Flour and malt	<5 mg/m^3^	38	4.36	4.5	1.14	7.0	2.0
Isocyanates	<20 ppb	29	13.98	15.0	3.6	22.5	7.0

The mean of all samples for a given concentration is used as the calibration point. Measurement equipment was calibrated independently for lactose.

Direct measurement during a 1.5-hour generation of N-butyl acetate (excluding the burn-in period) was used to calculate the average concentration for periods of five minutes. This gave 18 averages where the difference between the largest and smallest value was less then 3% (the mean of the averages was 52.1 mg/m^3 ^with standard deviation of 0.3 mg/m^3^, the maximum value was 52.7 mg/m^3 ^and the minimum value was 51.4 mg/m^3^). This implies that the average concentration over an interval of a few minutes is relatively constant any time in a generation.

## Specific Inhalation Challenges

Table 2 gives information on the targeted and obtained concentrations during the generation of lactose, flour and isocyanates. All products were generated from vials in which the available concentration was 50 mg/m^3^. The obtained concentrations were close to the targeted concentrations with small variability.

The GenaSIC apparatus was used for exposing 44 different subjects referred for possible OA (Table 3). In 37 instances, N-butyl acetate was used as the control agent. In 19 of these 37 subjects, the test consisted of a sole exposure to N-butyl acetate for 30 minutes. These subjects were subsequently exposed to potential causal agents (acrylates, isocyanates, etc.) using other methodologies if the tests were carried out in the hospital laboratory or in the workplace. In all other instances (n = 18), exposure to N-butyl acetate preceded exposure to formaldehyde (n = 8) or isocyanate (n = 10) with the GenaSIC.

**Table 3:** Characteristics of subjects with positive reactions

**No**.	Sex	Age (yrs)	FEV1 (% pred)	PC20 (mg/ml)	Agent	Duration of exposure (min)	Reaction
1	M	33	107	4.4	HDI	120	changes in PC20 and sputum cells
2	M	40	86	>32	malt	120	hypersensitivity-pneumonitis-like
3	M	27	83	0.06	wheat flour	60	immediate reaction; max. fall in FEV1: 24%
4	M	57	70	ND	HDI	120	late reaction; max. fall in FEV1: 27%
5	M	33	95	0.5	HDI	30	immediate reaction; max. fall in FEV1: 22%
6	M	49	90	0.5	wheat flour	90	immediate reaction; max. fall in FEV1: 27%
7	M	36	97	1.3	wheat flour	2.5	immediate reaction; max. fall in FEV1: 21%
8	M	26	101	1.3	wheat flour	1	immediate reaction; max. fall in FEV1: 20%
9	M	48	91	1.5	wheat flour	2	immediate reaction; max. fall in FEV1: 28%
10	M	44	100	0.4	TDI	20	late reactions; max. fall in FEV1: 51%

FEV1: forced expiratory volume-one second; PC20: concentration of methacholine causing a fall of 20% in FEV1

Eight different subjects were exposed to formaldehyde incrementally for 4, 30 and 120 minute periods. All these tests proved negative; i.e., with no significant (<10%) changes in FEV1 in the following minutes and hours. Ten other subjects were exposed to isocyanates (TDI in two instances and HDI in eight others). In six subjects, the test proved negative for up to two hours of exposure with GenaSIC and also, for two hours in a realistic way on a subsequent day. Four subjects had a positive reaction (Table 3); one immediate (maximum fall in FEV1 of 22%) and two late reactions (maximum falls in FEV1 of 17% and 51%). In the other positive test, the subject had significant changes in reactivity to methacholine and marked increases in total cell count and % of neutrophils after being exposed for two hours to HDI. Seven other subjects were exposed to dry particles; six being exposed to wheat flour and one to malt powder. Lactose that was aerosolized for 30 minutes at a mean concentration of 3.25 mg/m^3 ^(Table 3) represented the control exposure in all these subjects. Exposure to flour varied from 1 to 120 minutes at a mean concentration of 4.36 mg/m^3 ^and five of the six tests resulted in immediate falls in FEV1 that varied from 21 to 28% (Table 3). In the other negative instance, subsequent exposure to flour in a realistic way for 2 hours did not cause any significant changes in FEV1. There was one subject who reacted to malt powder, developing a hypersensitivity-pneumonitis-like reaction (with fever, leucocytosis and a maximum fall in forced vital capacity of 12%).

## Discussion

The purpose of specific inhalation challenges in a hospital laboratory is to reproduce a possible asthmatic reaction by exposing the worker to a potential causal agent present at work, by generating stable and safe concentrations of the product and eliciting an asthmatic reaction for which the fall in FEV1 is significant and acceptable (20 to 30% change in FEV1). The development of exposure apparatuses fulfilled these criteria.

We have previously shown that dry particles can be generated at lower and more constant concentrations with a generator than with the realistic approach in which a worker is simply asked to tip dry particles in a ventilated cubicle [6]. In the case of isocyanates, we also showed that the variation of concentrations during the exposure with a generator was less than what is documented in a ventilated cubicle [7]. Albeit for the advantage of offering the possibility of generating dry particles, vapor and aerosols with the same equipment, the new generator allows for exposing workers to low and stable concentrations. In the case of dry particles (flour and malt), the variability of concentrations was low (see Table 2). In the case of isocyanates, the variability of concentrations that we documented with the GenaSIC generator used in the current study (mean ± SD of 14.0 ± 3.6 ppb) is comparable to what we obtained with a previous generator (mean ± SD = 11.2 ± 3.9 ppb, range 4-24 ppb) [7].

The GenaSIC has several other advantages over the previous generation of close-circuit equipment. First, since the equipment is now commercialized, it is more easily accessible to the community interested in objective confirmation of occupational asthma and all scientific aspects related to human exposure to dry particles, vapor and aerosols. Secondly, the GenaSIC is less cumbersome than the previously described generators, and easier to clean. The smaller size of the equipment facilitates handling and transport. Also, the generation sequences are automated, which greatly reduces intervention on the part of technicians responsible for the challenges. The technician's target concentration is automatically set by the apparatus. Therefore, the method of exposure might be repeatable from one center to another using the same equipment. A more generalized use can greatly improve expertise and exchanges. The main limitation of the GenaSIC is, for the time being, the fact that it can generate only a limited quantity of occupational agents. Each new agent must be validated before exposure. However, flour and isocyanates that represent the most frequent causes of OA can be generated. The GenaSIC is also a highly technical device, requiring trained personnel on the operation and maintenance of device as well as clinical procedures. This limits its accessibility to centers having sufficient resources for such equipment. Use by untrained personnel can translate in malfunctions and improper results. Training for clinicians, technicians and medical engineers is offered with the device to mitigate this limitation.

Both cost (regional pricing for the GenaSIC can be obtained by contacting SCL Medtech through their website: http://www.sclmedtech.com) and handling time of the GenaSIC could also be seen as limitations to the widespread usage of such equipment. Indeed, the GenaSIC represents a significant investment and handling of the device before (preparation) and after (cleaning) patient exposure is somewhat time consuming. We however feel that in both cases these represent a significant improvement on realistic exposure methods. The costs of setting up a realistic exposure environment are those of the cubicle and exhaust system, which are significant and often unfeasible in hospital laboratories. The GenaSIC removes the need for a dedicated exhaust system because of integrated filters on the elimination line. Preparation of the GenaSIC for a specific challenge is estimated at 30 minutes, which is slightly superior to cubicle preparation. Cleaning of the GenaSIC requires 15 minutes of manipulation and 25 minutes of automated cleaning. Depending on the substance generated, this can be significantly less than the time required to clean a cubicle. Finally, the GenaSIC can also offer reduced costs when compared to custom made close-circuit equipments because of its compliance to electrical standards and its use of off-the-shelves components [6,7,14].

The main risk of specific inhalation challenges is related to the magnitude of the immediate reaction that occurs in the period up to 20 minutes following cessation of exposure [5] Therefore, it is important to control the exposure so as to obtain a progressive change in airway obstruction that does not exceed 30%, similar to what is generally aimed for in non-specific challenges. In a previous study, we showed that the use of generators resulted in a significantly reduced occurrence of exaggerated immediate reactions defined as changes in FEV1 that exceed 30% by comparison with the realistic approach [9]. In our study, we were able to obtain such acceptable reactions in all instances of immediate reactions. Exaggerated late reactions can still occur and although the risk can be reduced with generators, it was not significantly so in a previous study [9] and in the current study in which a late reaction with a fall of 51% in FEV1 occurred. These reactions are more difficult to predict. However, since they occur progressively over three to four hours, they are generally easily amenable to treatment. It is interesting to note that in the case of negative reactions using the GenaSIC methodology, subjects were also exposed in a realistic way. No subject showed asthmatic reactions with the realistic approach in which concentrations could only be monitored in the case of isocyanates. It has been shown that exposure in a realistic way results in higher and more variable concentrations than by using closed-circuit instruments [6,7]. Therefore, there were no instances of false negative reactions.

We need to validate the results of this study in a greater number of subjects by exposure to other potential occupational agents that exist as dry particles, vapor and wet aerosol. As mentioned above, expanding the use of such equipment may greatly contribute to a more precise diagnosis of OA, minimizing potential risks of unexpected asthmatic reactions.

## List of abbreviations

FEV1: forced-expiratory-volume one second; HDI: hexamethylene-diisocyanate; OA: occupational asthma; PC20: concentration of methacholine causing a fall of 20% in FEV1; TDI: toluene-diisocyanate.

## Competing interests

JLM holds no intellectual property and no commercial interest in the GenaSIC instrument. These are owned by SCL Medtech. SL and SC work for SCL Medtech (see title page).

This research work was supported by public organizations: The Canadian Institutes of Health Research (UOP - 77850 and the Center for Asthma in the Workplace, CDA66154) and the Ministère du Développement Économique, de l'Innovation et de l'Exportation du Québec (VT05-039) (see acknowledgements).

This work was not supported by the tobacco industry.

## Authors' contributions

SC was responsible for equipment and technical aspects. SB contributed to the study design, administrative duties and writing of the manuscript. JCB is a medical research student who contributed to data collection related to the equipment. JLM was responsible for collecting clinical data and writing of the manuscript. All authors have read and approved the final manuscript.
